# A Visual Attention Analysis Method for Industrial Patrol Inspection Based on Eye Tracking and Deep Learning

**DOI:** 10.3390/s26185817

**Published:** 2026-09-14

**Authors:** Wanqi Dai, Xuefei Li, Jinyi Fu, Xiubo Chen, Chao Liu, Sheng Miao

**Affiliations:** 1 School of Artificial Intelligence, Qingdao University of Technology, Qingdao 266520, China; dwq0117@gmail.com; 2 School of Environmental and Municipal Engineering, Qingdao University of Technology, Qingdao 266520, China; lixuefei@qut.edu.cn (X.L.); fujinyi0923@outlook.com (J.F.); liuchao@qut.edu.cn (C.L.); 3 Rizhao Chengtou Environment and Technology Group Company Limited, Rizhao 276500, China; xiubo_chen@hotmail.com

**Keywords:** industrial patrol inspection, visual attention, eye tracking, object detection, deep learning

## Abstract

Manual industrial patrol inspection relies heavily on on-site visual observation, while the visual attention of inspectors toward specific target equipment is difficult to quantify. This study proposes an industrial patrol inspection visual attention analysis method integrating eye tracking and deep learning-based object detection. A wearable eye-tracking device is used to acquire first-person inspection videos, eye-tracking data, and camera parameters. After temporal alignment of the multimodal data, the 3D gaze points are projected onto the corresponding 2D inspection video frames. YOLO12m is employed to detect target equipment, followed by frame-by-frame matching between the 2D gaze points and the equipment bounding boxes. Based on the matching results, seven visual attention metrics are calculated to quantitatively characterize visual attention during patrol inspection. On-site experiments with 10 participants yielded 21 inspection records, with overall visual attention scores ranging from 43.16 to 89.71 and averaging 75.99. Three experts independently rated the 21 records, and the system-generated ratings agreed with the majority expert ratings for 19 records. These results demonstrate that, under the present experimental conditions, the proposed method can quantify the visual attention of inspectors toward specific target equipment during industrial patrol inspection using multiple metrics and provide interpretable results.

## 1. Introduction

Industrial patrol inspection is an essential component of equipment operation and maintenance, typically requiring inspectors to follow prescribed routes and check the operating status of equipment and key inspection points [1]. Such inspections enable the timely identification of equipment abnormalities and potential faults, thereby reducing the risk of equipment failure and supporting equipment maintenance and management decisions [2]. Industrial environments often involve numerous pieces of equipment and complex operating conditions. Therefore, conducting on-site inspections carefully is important for stable equipment operation and production safety [3]. Manual inspection continues to play an important role in industrial settings. However, it relies on on-site observations by inspectors, introducing a degree of subjectivity [4].

The objective of industrial patrol inspection is to identify equipment abnormalities and potential defects in a timely manner [5]. Achieving this objective requires inspectors to observe the target equipment sufficiently and effectively, that is, to devote adequate visual attention to it [6]. Therefore, analyzing the visual attention of inspectors during patrol inspection helps provide information about their actual observation of the target equipment. Eye-tracking technology can record gaze information, providing data for analyzing visual attention [5]. Augmented reality head-mounted displays, such as Microsoft HoloLens 2, are equipped with integrated eye trackers and can capture eye-tracking data while users move and perform on-site tasks [7]. Deep learning-based object detection models can automatically localize objects in images and output their class labels and bounding boxes, providing the basis for equipment recognition in inspection videos [8]. In recent years, YOLO-based object detectors have been applied in industrial settings owing to their efficient inference and strong detection performance [9].

This study proposes an industrial patrol inspection visual attention analysis method integrating eye tracking and deep learning-based object detection. First-person inspection videos, eye-tracking data, and camera parameters are acquired using a wearable eye-tracking device. The multimodal data are first temporally aligned, after which the 3D gaze points are projected onto the 2D inspection video frames. YOLO12m is then employed to detect target equipment in the video and obtain the corresponding equipment classes and bounding boxes. Frame-by-frame matching is subsequently performed between the projected gaze points and the equipment bounding boxes. On this basis, the visual attention of inspectors toward specific target equipment is analyzed and quantified using the matching results. The major contributions of this study are as follows:

1. Eye-tracking data are utilized to characterize the visual attention of inspectors during patrol inspection, enabling objective and quantitative analysis of visual attention that is otherwise difficult to observe.
2. Deep learning-based object detection is applied to identify specific target equipment in the inspection scene, enabling differentiated analysis of visual attention for different target equipment.
3. By associating visual attention with specific target equipment, the visual attention of inspectors toward individual equipment items can be quantitatively analyzed using visual attention metrics.

## 2. Related Work

With the continued digital transformation of industrial operation and maintenance and the growing demand for intelligent inspection, research on digital and intelligent technologies for industrial patrol inspection has continued to advance, and various methods and systems have been developed. Jin and Tong developed a wearable-device-based intelligent patrol inspection system for power stations, enabling real-time collaboration between on-site inspectors and a remote diagnosis center [10]. Liu et al. developed a positioning system for large-scale indoor patrol inspection to record the locations and movement trajectories of inspectors [11]. Wang and Qi developed a multi-user collaborative augmented reality system to assist on-site personnel in inspecting laser-cutting equipment [12]. Peng et al. proposed an inspection route optimization method for natural gas stations that considers station priority, thereby providing intelligent decision support for inspection route planning [13]. Hesselink et al. developed a virtual-reality-enabled digital twin platform for sewage pumping station inspection, providing inspectors with an interactive environment for accessing equipment and sensor information [14]. Shao et al. developed a virtual inspection system with visual and haptic feedback, enabling operators to perform pumping station inspection tasks in a virtual environment [15]. Xu et al. developed a wearable augmented reality system for mechanical equipment inspection to provide inspectors with equipment status and maintenance information [16]. Tian et al. proposed a rail transit equipment inspection system based on 5G and edge intelligence, enabling integrated monitoring of inspectors and equipment conditions during inspection [17]. Existing studies have primarily employed digital and intelligent technologies to improve task assistance and process management in industrial patrol inspection, while relatively limited attention has been paid to the visual attention of inspectors toward target equipment during actual patrol inspection and its quantitative characterization.

Deep learning-based object detection has been widely applied in industrial scenarios to automatically locate equipment, components, and defect regions. Im and Jeong applied an R-CNN-based object detection method to laser-cutting quality inspection of large automotive components, enabling the automatic localization of cutting positions and defect regions [18]. Oh et al. applied Faster R-CNN to radiographic image analysis of ship welds, enabling the automatic identification of welding defect locations and types [19]. Lian et al. applied Mask R-CNN to automated visual inspection of printed circuit boards, achieving the localization and segmentation of surface-mounted components [20]. Yang et al. applied SSD to tiny-component detection on manufacturing production lines, enabling the real-time detection and identification of defective components [21]. Yang and Liu applied RetinaNet to steel surface quality inspection, achieving the automatic localization and classification of different types of steel surface defects [22]. Çelik et al. applied YOLOv8 to the inspection of automotive plastic components, enabling the real-time detection of surface defects [23]. Zhang et al. used object detection to identify valves and determine their position states, enabling the real-time monitoring of abnormal valve positions in industrial environments [24]. Bao et al. applied object detection to transmission-line inspection images, enabling the automatic identification of anomalies in components such as insulators and vibration dampers [25]. The above studies demonstrate that deep learning-based object detection has shown good applicability in a variety of industrial scenarios, providing important technical support for the automation and intelligent development of industrial inspection and monitoring.

Eye-tracking technology has been widely applied across diverse task contexts to investigate visual attention. Li et al. used eye tracking to compare expert–novice differences in attentional processes during simulated surgical tasks [26]. Wang et al. used eye tracking during driving tasks to investigate the effects of emotional states and cognitive load on the visual attention of drivers [27]. Hong et al. used eye-tracking experiments to examine the influence of different visual environments on hazard recognition among construction equipment operators [28]. Aust et al. used eye tracking to investigate the visual inspection of aircraft engine blades and examined the effect of blade cleanliness on inspection performance [29]. Ulutas et al. used eye tracking to analyze the visual activities of quality control operators in a manufacturing facility and compare experienced and novice operators [6]. Saleem et al. analyzed the gaze patterns of inspectors during facade inspection to investigate their visual attention and sense-making processes [30]. Takamido et al. analyzed the visual attention and head positioning behavior of experts and knowledgeable novices during simulated refinery patrol inspection [5]. Cho et al. integrated eye tracking with deep learning-based object detection to determine which parts of inspected objects were observed by workers and analyze their gaze behavior during manufacturing quality inspection [31]. These studies demonstrate that eye-tracking technology can be effectively applied to the analysis of visual attention in inspection-related tasks, and that combining it with object detection to establish associations between gaze data and inspected objects is also feasible. However, in industrial patrol inspection scenarios, how to further integrate eye tracking with deep learning-based object detection to establish associations between the visual attention of inspectors and specific target equipment, and to quantitatively analyze the visual attention of inspectors toward different target equipment, has not yet been sufficiently addressed in existing industrial patrol inspection studies.

## 3. Methodology

### 3.1. Inspection Scenario and Workflow

**Figure 1:**
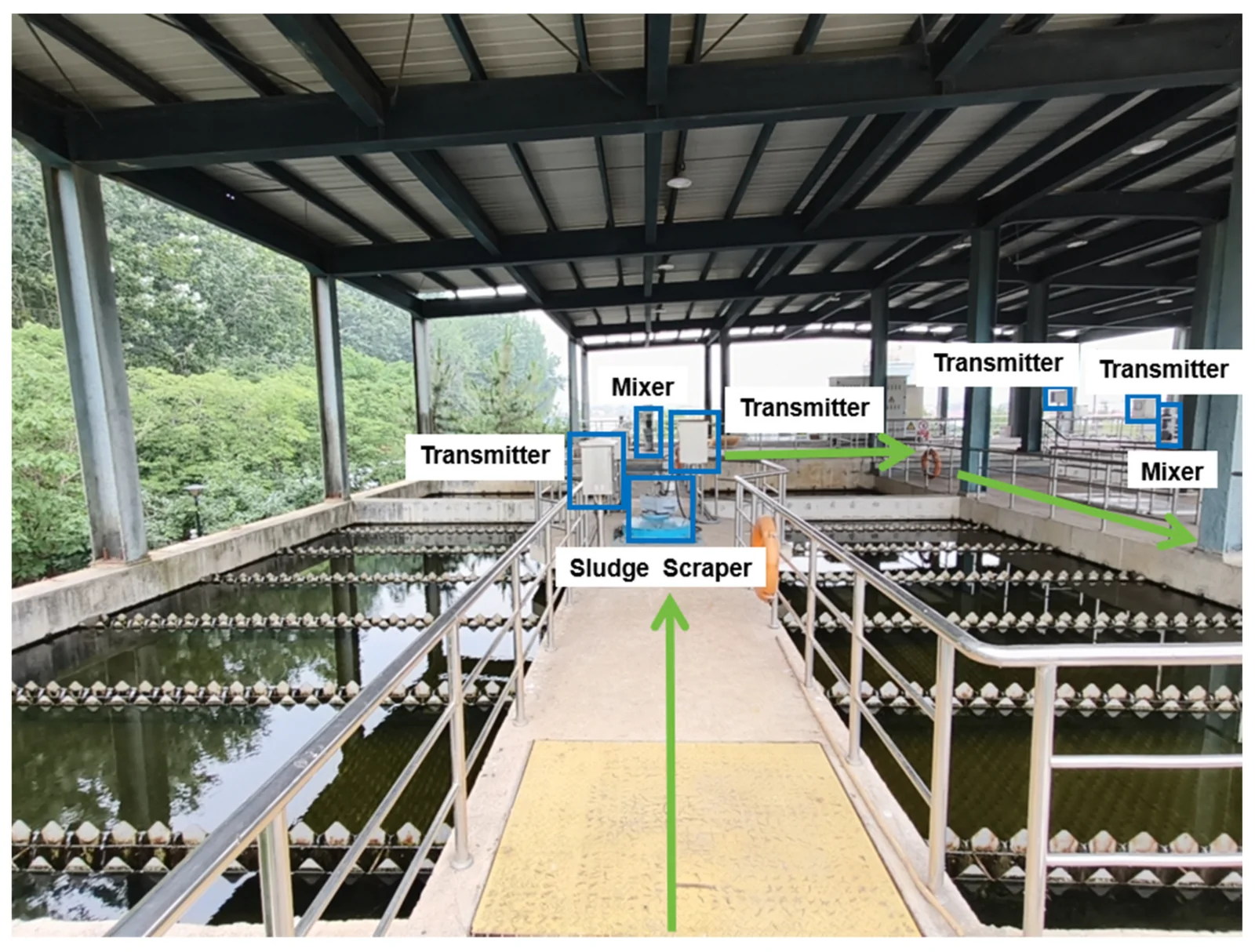
Inspection scenario in the high-density sedimentation tank area.

The inspection scenario was a high-density sedimentation tank area at a wastewater treatment plant, as shown in Figure 1. The high-density sedimentation tank is an important structure in the wastewater treatment process. Various types of equipment are distributed throughout the area, including valves, transmitters, mixers, flowmeters, and sludge scrapers. The selected equipment set includes several common equipment types, particularly valves, transmitters, mixers, and flowmeters, that are widely used in industrial facilities beyond wastewater treatment plants. Given the large number of equipment items, the complex spatial layout, and the relatively fixed inspection route, inspectors were required to follow the prescribed route and sequentially inspect each key equipment item, making the area suitable for analyzing the visual attention of inspectors toward multiple target equipment items during patrol inspection.

**Figure 2:**
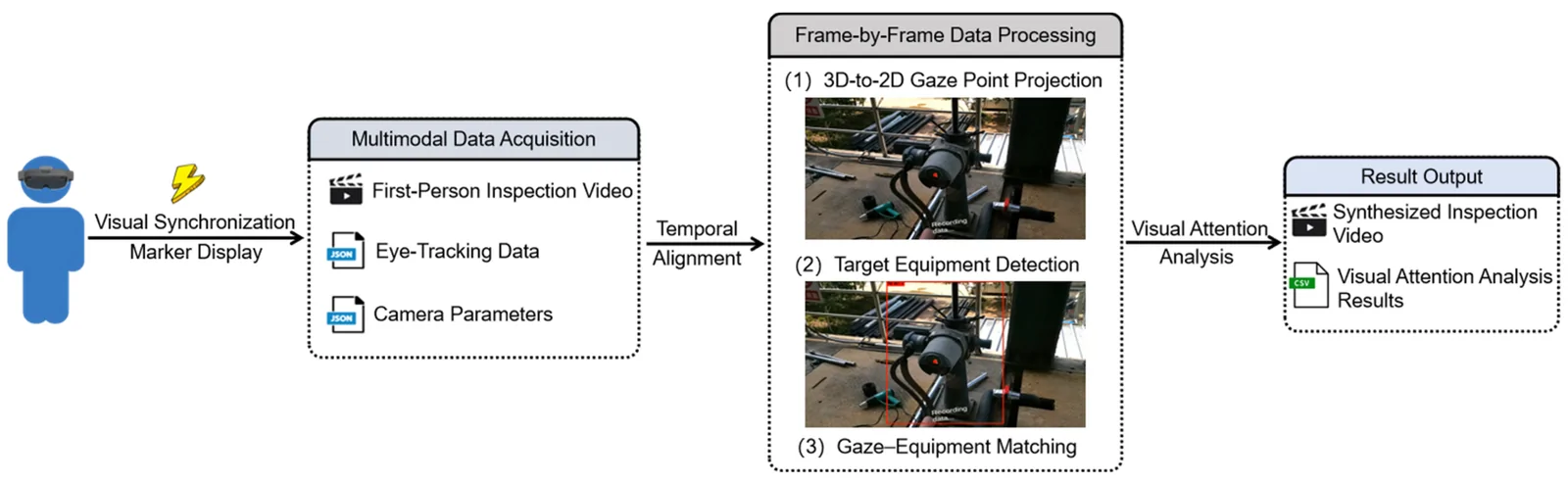
Workflow of the proposed industrial patrol inspection visual attention analysis method.

The workflow of the proposed method is shown in Figure 2. In the high-density sedimentation tank inspection scenario, the inspector wearing a wearable eye-tracking device begins the patrol inspection after the visual synchronization marker disappears. During the inspection, the device simultaneously acquires the first-person inspection video, eye-tracking data, and camera parameters. After the inspection is completed, the acquired multimodal data are first temporally aligned using the visual synchronization marker. The data are then processed frame by frame, primarily by projecting the 3D gaze points onto the corresponding 2D video frames and detecting the target equipment using a deep learning-based object detection model. Based on the matching results between the 2D gaze points and the equipment bounding boxes, the system quantitatively analyzes the visual attention of inspectors toward different target equipment during patrol inspection and outputs a synthesized inspection video and visual attention analysis results.

### 3.2. System Architecture

**Figure 3:**
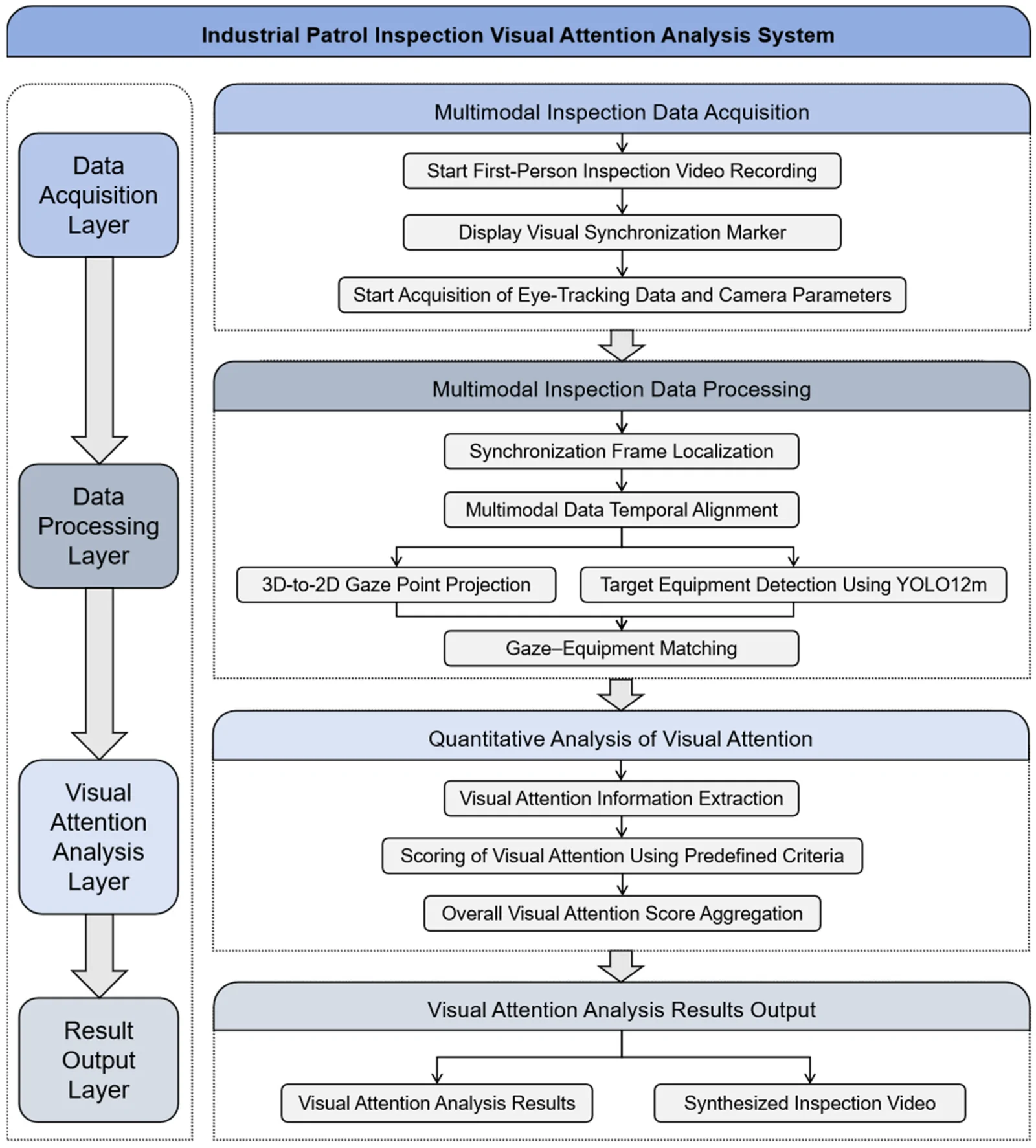
Architecture of the industrial patrol inspection visual attention analysis system.

The industrial patrol inspection visual attention analysis system comprises four layers: the data acquisition layer, the data processing layer, the visual attention analysis layer, and the result output layer, as illustrated in Figure 3. In the data acquisition layer, once the recording of the first-person inspection video begins, the system displays a visual synchronization marker within the field of view of the inspector for the subsequent temporal alignment of the video and sensor data. After the visual synchronization marker disappears, the system simultaneously starts acquiring the eye-tracking data and camera parameters while continuing to record the first-person inspection video, thereby enabling parallel acquisition of the three types of data.

In the data processing layer, the system first locates the first frame after the visual synchronization marker disappears and designates it as the synchronization frame. This establishes a temporal mapping from the video timeline to the sensor timeline, thereby completing the temporal alignment of the multimodal data. On this basis, the system uses the camera parameters to project each 3D gaze point onto the corresponding video frame, yielding a 2D gaze point. Meanwhile, the YOLO12m object detection model is employed to detect the target equipment in the video frames. YOLO12m balances detection accuracy and inference efficiency, making it suitable for equipment detection in first-person inspection videos with complex backgrounds and varying equipment scales. The system then determines, on a frame-by-frame basis, whether each 2D gaze point falls within any equipment bounding box. A gaze point is considered matched to an equipment item if it falls within the corresponding equipment bounding box; otherwise, it is considered unmatched. Through this matching process, associations between gaze information and specific target equipment are established.

In the visual attention analysis layer, visual attention toward the target equipment is analyzed based on the matching results. The corresponding visual attention metrics are then calculated and scored according to predefined criteria. After all metrics have been calculated and scored, the resulting scores are aggregated to obtain an overall visual attention score. The individual metrics quantitatively characterize different aspects of visual attention, while the overall visual attention score is a task-specific composite score calculated from the seven predefined metrics to summarize visual attention under the inspection conditions considered in this study.

In the result output layer, the system outputs visual attention analysis results and a synthesized inspection video. The visual attention analysis results include the scores for each visual attention metric, the overall visual attention score, and the corresponding scoring basis. The synthesized inspection video overlays the equipment bounding boxes and 2D gaze points onto the original first-person inspection video to support inspection process replay.

### 3.3. Data Acquisition Device

**Figure 4:**
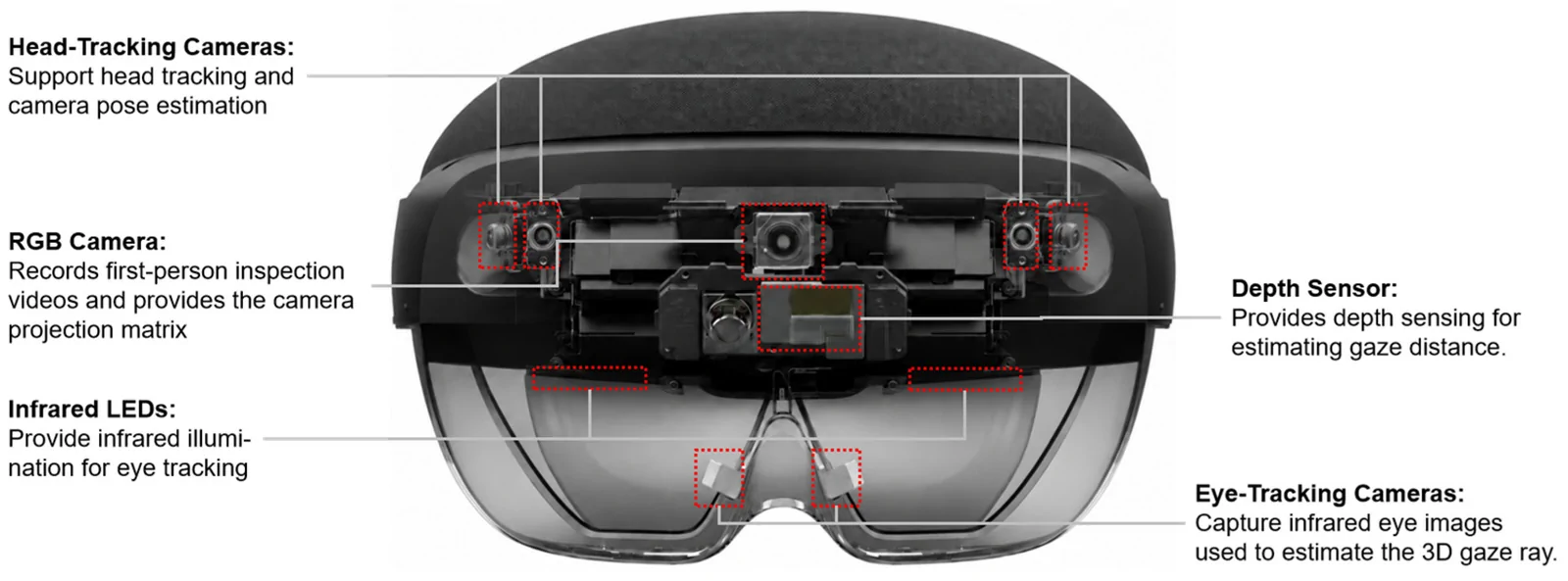
Locations and functions of the main hardware components of Microsoft HoloLens 2.

Microsoft HoloLens 2 (Microsoft Corporation, Redmond, WA, USA) was used as the wearable eye-tracking device in this system. HoloLens 2 integrates multiple hardware components, including an RGB camera, eye-tracking cameras, infrared LEDs, a depth sensor, and head-tracking cameras, providing capabilities for first-person inspection video recording, eye tracking, and head tracking. The locations and functions of its main hardware components are shown in Figure 4. The data acquisition application running on HoloLens 2 was developed using Unity3D (version 2021.3.45f2c1) and deployed as a Universal Windows Platform (UWP) application, with Windows 10 SDK 10.0.19041.0 specified as the target SDK. In addition, Mixed Reality Toolkit (MRTK) 2.8.3 was integrated to provide access to HoloLens 2 capabilities, including eye-gaze input and spatial awareness.

Before formal data acquisition, each participant first completed the eye-tracking calibration using the built-in calibration function of HoloLens 2. During calibration, the participant followed the system instructions and sequentially fixated on the displayed holographic calibration targets, allowing the device to perform user-specific eye-tracking calibration based on the individual eye characteristics. HoloLens 2 stores the corresponding calibration information locally on the device and automatically applies the calibration information associated with the current user during eye tracking, thereby accommodating individual differences in eye characteristics and reducing their influence on eye-tracking accuracy.

During each inspection, the device acquires three types of data: first-person inspection video, eye-tracking data, and camera parameters. The first-person inspection video records the scene viewed by the inspector and is captured at 30 frames per second (fps). The eye-tracking data are sampled at approximately 30 Hz and mainly include the gaze origin, gaze direction, and gaze distance. The gaze distance is obtained from the HoloLens 2 spatial sensing system based on the intersection between the gaze ray and the reconstructed spatial environment. The gaze origin, gaze direction, and gaze distance jointly determine the corresponding 3D gaze point in the spatial coordinate system. The camera parameters include the camera projection matrix and the camera pose matrix. The camera projection matrix describes the camera projection relationship, whereas the camera pose matrix describes the spatial transformation from the camera coordinate system to the world coordinate system.

### 3.4. Eye-Tracking Data Processing

To project the 3D gaze data onto the first-person inspection video frames, the inspection video, eye-tracking data, and camera parameters were first temporally aligned. Because these three types of data were acquired through separate data streams, the video playback time did not directly correspond to the timestamps associated with the eye-tracking data and camera parameters. Therefore, a common temporal origin was established. After the video recording began, a visual synchronization marker was displayed within the field of view of the inspector and captured in the inspection video. Once the marker disappeared, the recording of the eye-tracking data and camera parameters began simultaneously. Accordingly, the first video frame after the marker disappeared was defined as the initial frame of the valid inspection video and temporally aligned with the first eye-tracking record and the first set of camera parameters. This frame is denoted by $f_{sync}$, and its corresponding video playback time is denoted b $t_{sync}$. The timestamp of the first eye-tracking record is denoted by $t_{base}$ and serves as the origin of the sensor timeline. During frame-by-frame processing, let $t_{msec}$ denote the playback time of the current video frame. The corresponding timestamp $t_{cur}$ on the sensor timeline is calculated using Equation (1).

(1) $$t_{cur}=t_{base}+\left(t_{msec}-t_{sync}\right)\times 10^{6}$$

where $t_{base}$ and $t_{cur}$ are expressed in nanoseconds, whereas $t_{msec}$ and $t_{sync}$ are expressed in milliseconds. Therefore, the time difference is multiplied by 10^6^ to convert milliseconds to nanoseconds. After $t_{cur}$ is obtained, the eye-tracking record and the set of camera parameters with timestamps closest to $t_{cur}$ are separately identified and assigned to the current video frame. During eye-tracking data processing, samples with invalid gaze origin, gaze direction, or gaze distance values, including those caused by temporary eye-tracking loss such as eye closure or failure to detect eye features, were excluded without interpolation. No additional blink detection algorithm was applied in this study.

After temporal alignment of the multimodal data, the 3D gaze points are first calculated from the eye-tracking data and then projected onto the corresponding 2D video frames. Let o and d denote the gaze origin and gaze direction in the world coordinate system, respectively. Their vector forms are given by Equations (2) and (3), and the corresponding 3D gaze point $P_{world}$ is calculated using Equation (4).

(2) $$o={\left[o_{x},o_{y},o_{z}\right]}^{T}$$

(3) $$d={\left[d_{x},d_{y},d_{z}\right]}^{T}$$

(4) $$P_{world}=o+\lambda d$$

where $o_{x}$, $o_{y}$, and $o_{z}$ denote the three components of the gaze origin in the world coordinate system, $d_{x}$, $d_{y}$, and $d_{z}$ denote the three components of the gaze direction in the world coordinate system, λ denotes the gaze distance, $P_{world}$ denotes the resulting 3D gaze point in the world coordinate system, and the superscript T denotes the transpose operation.

Because the 3D gaze point is expressed in the world coordinate system, whereas the camera projection matrix maps points in the camera coordinate system to clip space, the gaze point must first be transformed into the camera coordinate system. Let $M_{c2w}$ denote the camera pose matrix describing the transformation from the camera coordinate system to the world coordinate system and let $M_{proj}$ denote the camera projection matrix. The inverse matrix $M_{c2w}^{-1}$ therefore represents the transformation from the world coordinate system to the camera coordinate system. After the 3D gaze point is represented in homogeneous coordinates, its camera-coordinate representation ${\tilde{P}}_{cam}$ is calculated using Equation (5). It is then mapped to clip space using Equation (6) to obtain ${\tilde{P}}_{clip}$.

(5) $${\tilde{P}}_{cam}=M_{c2w}^{-1}\left[\begin{array}{c} P_{world}\\ 1 \end{array}\right]$$

(6) $${\tilde{P}}_{clip}=M_{proj}{\tilde{P}}_{cam}=\left[\begin{array}{c} x_{clip}\\ y_{clip}\\ z_{clip}\\ w_{clip} \end{array}\right]$$

where the tilde denotes the homogeneous-coordinate representation of a point, and $x_{clip}$, $y_{clip}$, $z_{clip}$, and $w_{clip}$ denote the four components of the homogeneous coordinates in clip space.

The normalized device coordinates $x_{ndc}$ and $y_{ndc}$ are obtained through perspective division using Equation (7). Finally, the normalized device coordinates are converted into the 2D pixel coordinates u and v in the corresponding video frame using Equation (8).

(7) $$\left[\begin{array}{c} x_{ndc}\\ y_{ndc} \end{array}\right]=\frac{1}{w_{clip}}\left[\begin{array}{c} x_{clip}\\ y_{clip} \end{array}\right]$$

(8) $$\left[\begin{array}{c} u\\ v \end{array}\right]=\left[\begin{array}{c} \frac{x_{ndc}+1}{2}W\\ \frac{1-y_{ndc}}{2}H \end{array}\right]$$

where W and H denote the video-frame width and height, respectively.

### 3.5. Visual Attention Quantification Criteria and Scoring Method

Before the formal experiments, the visual attention metrics, the score allocation for each metric, and the scenario-specific parameter settings were designed and adjusted through structured consultation with industrial inspection experts familiar with the inspection scenario and target equipment, together with preliminary inspection trials. Seven visual attention metrics were ultimately defined: Inspection Attentiveness (S1), Effective Inspection Time Ratio (S2), Target Equipment Observation Rate (S3), Equipment Observation Duration Compliance (S4), Gaze Stability (S5), Visual Scanning Coverage (S6), and Viewing Distance Appropriateness (S7). A 100-point scoring scheme was used to aggregate these seven predefined metrics into a task-specific overall visual attention score for the inspection scenario considered in this study. For qualitative interpretation of the overall score, three visual attention levels were predefined, with low (L) for scores below 60, moderate (M) for scores from 60 to below 85, and high (H) for scores of 85 or above. The two classification thresholds were established before the expert evaluation based on expert recommendations and preliminary inspection trials.

The metrics and corresponding score allocation are summarized in Table 1. The final parameter settings were determined by considering expert recommendations, the preliminary trial results, and the applicability of the parameters to the actual inspection conditions. These settings included the 4-s threshold and 5-point deduction for an attention-lapse event, the equipment observation duration thresholds, the viewing-distance thresholds, the gaze-stability criteria, and the required number of covered grid cells. S4–S7 were assigned equal weights because these four metrics characterize different and complementary aspects of visual attention toward individual equipment items, and no consistent basis was identified during the expert consultation for assigning greater importance to any one of them. The above metrics, score allocation, and parameter settings were established for the specific inspection scenario considered in this study and are not intended as universal criteria for industrial patrol inspection.

Based on the frame-by-frame matching results, consecutive gaze samples associated with the same target equipment item are treated as a continuous equipment observation segment. A segment lasting at least 200 milliseconds is regarded as an effective equipment observation segment in this study. This duration threshold was selected with reference to the temporal thresholds used in previous eye-tracking research and was applied in this study to define effective equipment observation segments [32]. The first three metrics characterize the overall visual attention of inspectors during the inspection process and are assigned 15 points each.

Inspection Attentiveness (S1) characterizes the continuity of visual attention toward the target equipment being observed. For S1, an attention-lapse event is operationally defined as a period of more than 4 consecutive seconds during which the gaze point remains outside all target equipment bounding boxes after leaving the bounding box of the currently observed equipment item. Five points are deducted for each event, with a minimum score of 0 for this metric. Effective Inspection Time Ratio (S2) characterizes the proportion of the total inspection time devoted to equipment inspection. A video frame is classified as an effective inspection frame when at least one target equipment item is visible in the frame. The durations of all effective inspection frames are accumulated, and the score is calculated based on the ratio of effective inspection time to the total inspection time. For Target Equipment Observation Rate (S3), a target equipment item is considered to have been observed when at least one effective equipment observation segment is identified for that item. The score is calculated based on the proportion of observed target equipment items among all target equipment items. The scores for S1, S2, and S3 are calculated using Equations (9), (10) and (11), respectively.

(9) $$S_{1}=\max\left(15-5N_{a},0\right)$$

(10) $$S_{2}=15\frac{T_{e}}{T}$$

(11) $$S_{3}=15\frac{N_{c}}{N_{t}}$$

where $N_{a}$ denotes the number of attention-lapse events, $T_{e}$ denotes the accumulated duration of effective inspection frames, T denotes the total valid inspection duration, $N_{c}$ denotes the number of target equipment items for which at least one effective equipment observation segment is identified, and $N_{t}$ denotes the total number of target equipment items.

The remaining 55 points are distributed equally among the 11 target equipment items, giving 5 points per equipment item. For each equipment item, the allocated score is further divided equally among S4–S7, with 1.25 points assigned to each metric. If no effective equipment observation segment is identified for an equipment item, the equipment item is considered unobserved, and S4–S7 for that item are all assigned 0 points.

Equipment Observation Duration Compliance (S4) characterizes whether the cumulative effective equipment observation duration for a target equipment item reaches the prescribed threshold for that item. The cumulative effective equipment observation duration $T_{i}$ for equipment item i is calculated using Equation (12).

(12) $$T_{i}=\sum t_{j}$$

where $t_{j}$ denotes the duration of the effective equipment observation segment j associated with the equipment item. Considering the differences in inspection content, the required cumulative equipment observation duration is set to 6 s for transmitters and flowmeters, for which instrument readings need to be observed in addition to operating status and external condition, and to 4 s for valves, mixers, and sludge scrapers, for which the inspection mainly involves operating status and external condition. The full S4 score is awarded when $T_{i}$ reaches the corresponding threshold; otherwise, the score is 0.

Gaze Stability (S5) characterizes the short-term spatial stability of the gaze trajectory during effective observation of a target equipment item. To reduce the influence of differences in equipment size and image scale, the horizontal and vertical gaze coordinates are first normalized according to the width and height of the corresponding equipment bounding box. The gaze samples within effective equipment observation segments are then divided into windows of 0.5 s. Given the approximately 30 Hz sampling rate of the HoloLens 2 eye tracker [7], each window normally contains approximately 15 gaze samples, allowing short-term gaze variation to be characterized while preserving temporal locality. For each window, gaze dispersion D is calculated using Equation (13).

(13) $$D=\sqrt{\sigma_{x}^{2}+\sigma_{y}^{2}}$$

where $\sigma_{x}$ and $\sigma_{y}$ denote the standard deviations of the normalized horizontal and vertical gaze coordinates within the window, respectively. A window is classified as stable when D≤0.10. Let $N_{s}$ denote the number of stable windows and N denote the total number of valid windows. The proportion of stable windows $R_{s}$ is calculated using Equation (14).

(14) $$R_{s}=\frac{N_{s}}{N}$$

The full S5 score is awarded when $R_{s}\geq 0.80$; otherwise, the score is 0. The dispersion threshold and the required proportion of stable windows were determined based on the preliminary trials and evaluated through expert consultation.

Visual Scanning Coverage (S6) characterizes the adequacy of visual observation over the front-facing two-dimensional region of a target equipment item. A grid-based approach has previously been used in eye-tracking research to quantify the spatial distribution of visual attention by dividing the viewed region into cells and determining the regions receiving gaze samples [33]. Following this principle, the bounding box of each target equipment item is divided into a 3 × 3 virtual grid in this study. For each grid cell, the observation time associated with gaze samples within effective equipment observation segments that fall within the cell is accumulated, and a cell is considered covered when the accumulated duration reaches at least 200 milliseconds. The full S6 score is awarded when at least five of the nine cells are covered. The five-cell criterion requires visual attention to be distributed over more than half of the predefined equipment regions rather than remaining concentrated in only a small local region.

Viewing Distance Appropriateness (S7) characterizes whether target equipment observation occurs within the predefined maximum viewing distance. In this study, the viewing distance is represented by the gaze-distance value associated with each gaze sample within an effective equipment observation segment. Based on the inspection contents and the expert consultation, transmitters and flowmeters, for which instrument readings must be observed, use a maximum acceptable viewing distance of 1.5 m, whereas valves, mixers, and sludge scrapers use a maximum acceptable viewing distance of 2.0 m. Let $N_{d}$ denote the number of gaze samples within effective equipment observation segments that satisfy the corresponding maximum viewing-distance threshold, and let N denote the total number of gaze samples within those segments for the equipment item. The proportion of samples within the acceptable viewing distance $R_{d}$ is calculated using Equation (15).

(15) $$R_{d}=\frac{N_{d}}{N}$$

The full S7 score is awarded when $R_{d}\geq 0.80$; otherwise, the score is 0. The use of a proportion-based criterion reduces the influence of occasional fluctuations in gaze-distance measurements on the S7 score.

## 4. Experiments

All experiments were conducted in a Windows environment using an NVIDIA GeForce RTX 4060 Laptop GPU (NVIDIA Corporation, Santa Clara, CA, USA). The software environment was based on Python 3.11.15. Model training, validation, and inference were performed using Ultralytics 8.4.37, with PyTorch 2.5.1 providing the underlying deep learning computation framework. OpenCV 4.13.0 was used for video processing and synthesized inspection video generation, while NumPy 2.4.3 was used for the matrix operations and numerical computations required for 3D gaze point projection.

### 4.1. Eye-Tracking Experiment

**Figure 5:**
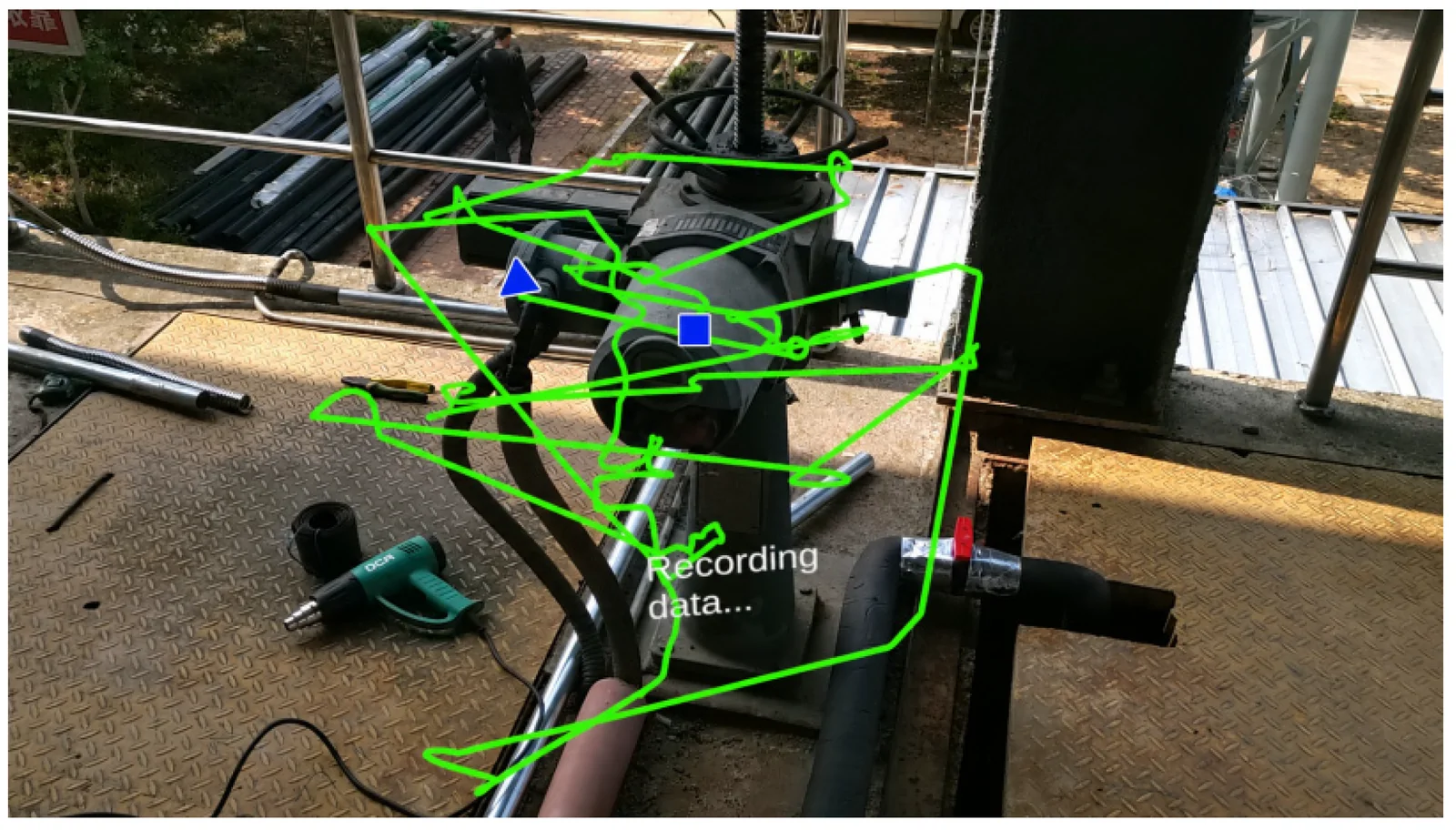
Visualization of the gaze trajectory on the inspection video. The green curve represents the gaze trajectory, while the blue square and triangle indicate the start and end points, respectively.

To demonstrate the feasibility of eye-tracking data acquisition and 3D-to-2D gaze point projection in the inspection scenario, eye-tracking data collected over a continuous time interval during which the inspector observed a valve were selected and temporally aligned with the corresponding first-person inspection video frames and camera parameters. Using the eye-tracking data processing method described above, the 3D gaze points were projected onto the 2D video frames and connected in temporal order to form a gaze trajectory, as shown in Figure 5. The green curve represents the trajectory of the projected 2D gaze points across the video frames during this time interval. The square indicates the start point of the gaze trajectory, and the triangle indicates the end point. It should be noted that the subsequent visual attention analysis is based on the 2D gaze points obtained frame by frame and their matching results with the equipment bounding boxes. The gaze trajectory presented here is used only as a visual illustration of the acquired eye-tracking data and the results of 3D-to-2D gaze point projection.

### 4.2. Modeling

This study constructed a target equipment detection dataset using on-site data acquired in the high-density sedimentation tank area of the wastewater treatment plant. The data consisted of first-person inspection videos recorded using HoloLens 2 and smartphones. After video frame extraction, image screening, and manual annotation, the dataset contained 995 images and 1395 annotated bounding boxes covering five equipment classes: valves, transmitters, mixers, flowmeters, and sludge scrapers. The number of annotated bounding boxes for each target equipment class is summarized in Table 2. Considering the high visual similarity between adjacent frames extracted from the same video, source videos were used as the grouping units for dataset partitioning rather than randomly assigning individual frames. All frames extracted from the same source video were assigned exclusively to one subset, ensuring that no source video was shared among the training, validation, and test sets. The resulting training, validation, and test sets contained 662, 163, and 170 images, respectively, with 906, 228, and 261 annotated bounding boxes. Each subset included all five equipment classes.

After the dataset was partitioned, the YOLO12m model was trained on the target equipment detection dataset. The input image size was set to 960 × 960 pixels, the maximum number of training epochs to 120, and the batch size to 3. AdamW was used as the optimizer. Early stopping with a patience of 25 epochs was employed, and training was terminated at epoch 105 because the validation metric did not improve for 25 consecutive epochs. To improve the robustness of the model to variations in illumination, viewpoint, and object scale, lightweight data augmentation was applied, including HSV augmentation, random translation and scaling, small-angle rotation and shear, perspective transformation, and Mosaic augmentation. The probability of Mosaic augmentation was set to 0.3.

**Figure 6:**
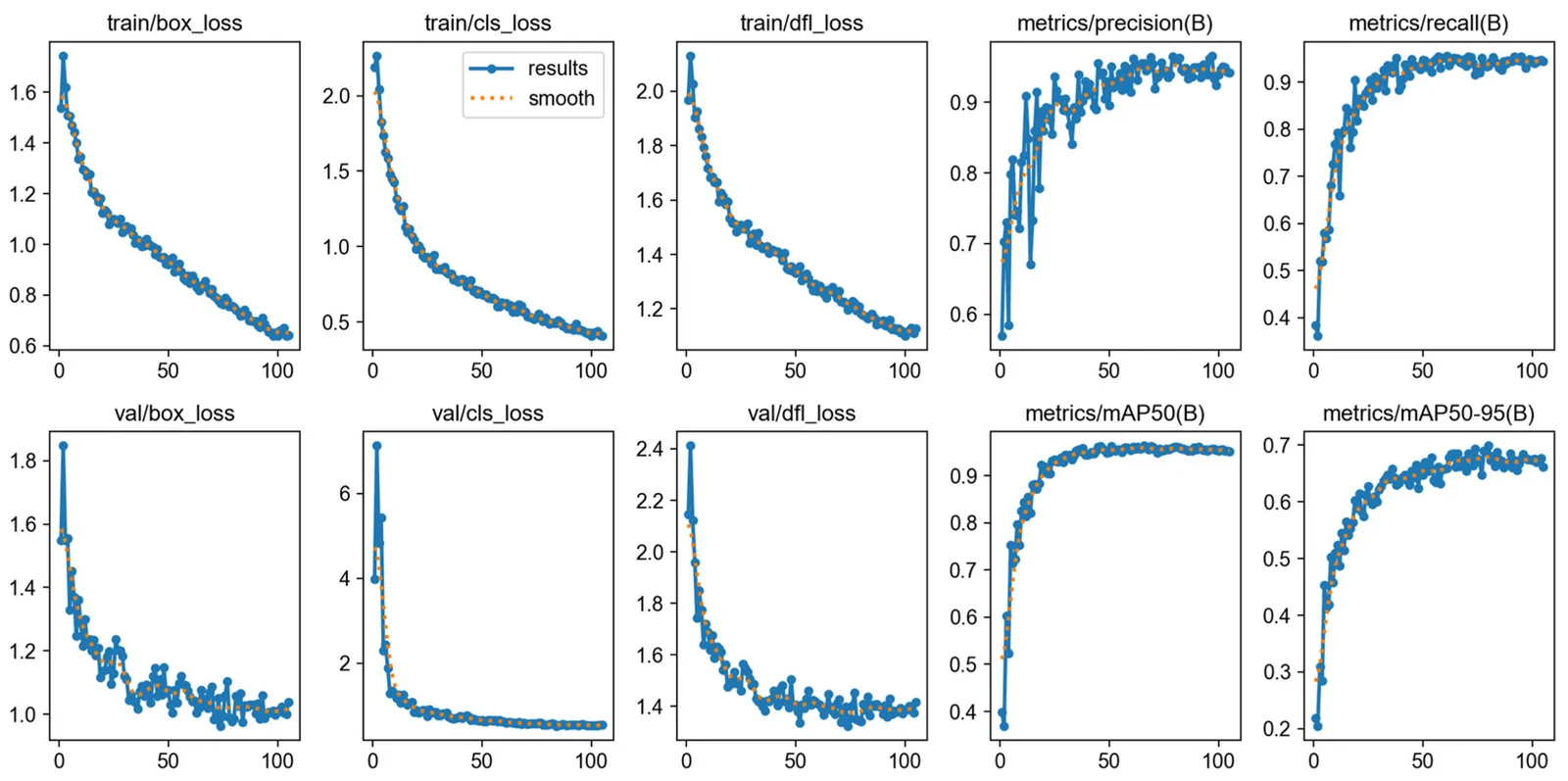
Training and validation curves of the YOLO12m model.

The training and validation curves are shown in Figure 6. As training progressed, the bounding-box regression loss, classification loss, and distribution focal loss on the training set decreased continuously, indicating progressive improvement in object localization and classification. The corresponding validation losses also exhibited overall downward trends and gradually stabilized during the later training epochs, suggesting stable convergence on the validation data. According to the training results, the model achieved its best validation performance at epoch 80, with a precision of 0.9583, a recall of 0.9346, an mAP@0.5 of 0.9603, and an mAP@0.5:0.95 of 0.6987.

After training, the model was evaluated on the independent test set, which was not involved in model training or model selection. The model achieved an overall precision of 0.9411, a recall of 0.9712, an mAP@0.5 of 0.9790, and an mAP@0.5:0.95 of 0.6992. Class-specific detection performance was further evaluated, as shown in Table 3.

**Figure 7:**
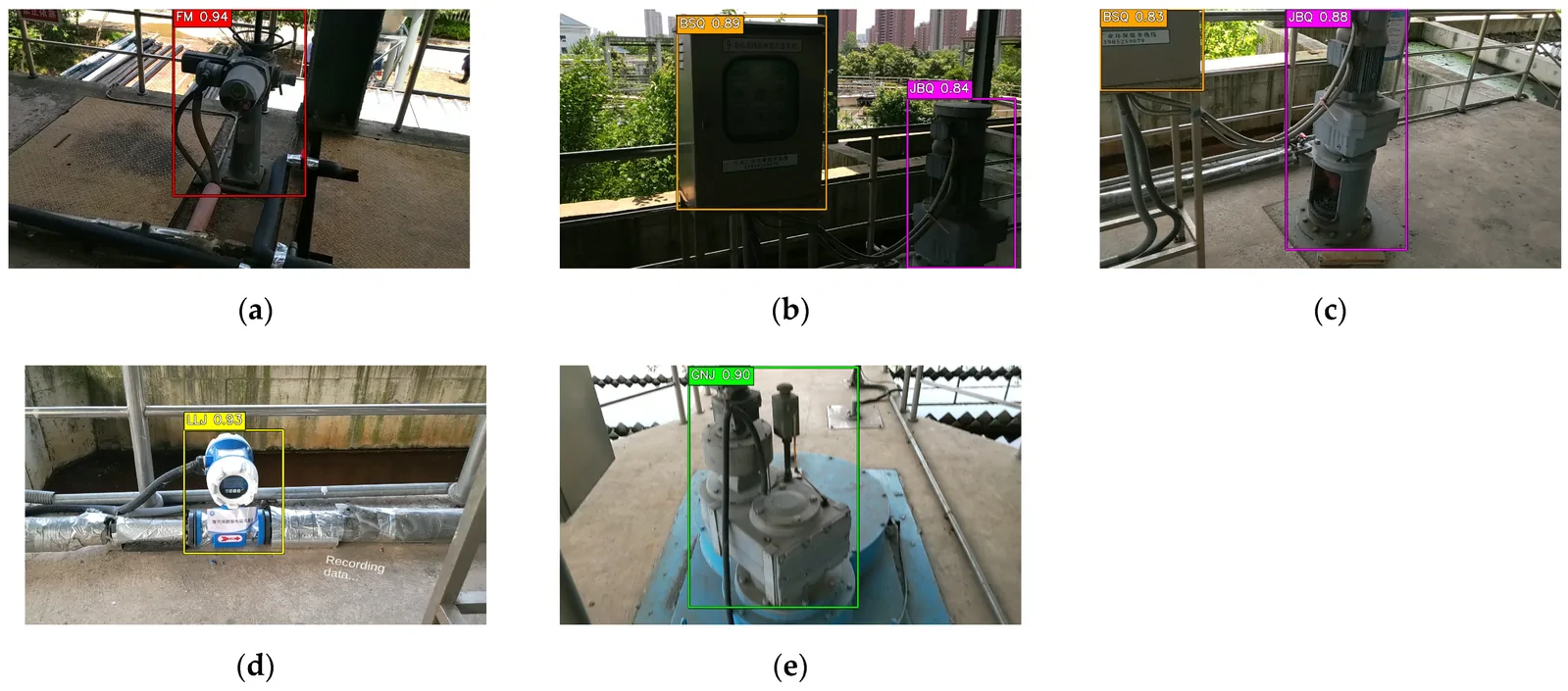
Representative detection results for the five target equipment classes on the independent test set: (**a**) valve; (**b**) transmitter; (**c**) mixer; (**d**) flowmeter; and (**e**) sludge scraper. The non-English text visible in the images is part of the original equipment labels in the inspection environment.

All five equipment classes achieved satisfactory detection performance on the independent test set, as summarized in Table 3. The AP@0.5 values ranged from 0.9493 to 0.9950, with valves, transmitters, mixers, flowmeters, and sludge scrapers achieving 0.9950, 0.9773, 0.9909, 0.9825, and 0.9493, respectively. The recall values for all five classes exceeded 0.93. Representative detection results for the five target equipment classes on the independent test set are shown in Figure 7.

### 4.3. Visual Attention Analysis Experiments

In May 2026, inspection experiments were conducted along the predefined route covering 11 target equipment items across five equipment classes in the high-density sedimentation tank area of the wastewater treatment plant. A total of 10 participants took part in the experiments. Based on their practical industrial inspection experience and familiarity with the inspection area and target equipment, the participants were categorized into three experience levels: high, moderate, and low. One participant had extensive practical industrial inspection experience and was familiar with the inspection area and target equipment; three participants had some practical industrial inspection experience and a certain degree of familiarity with the inspection area and target equipment; and the remaining six participants had limited practical industrial inspection experience and were relatively less familiar with the inspection area and target equipment. Each participant completed two or three inspection rounds, yielding a total of 21 inspection records. No specific inspection behaviors were deliberately induced or prescribed for testing purposes; participants completed the predefined inspection task without being instructed to exhibit particular behavior patterns. The experience level and corresponding inspection records for each participant are presented in Table 4.

To further describe the inspection content of the experiment, the predefined inspection route covered 11 target equipment items, and the corresponding inspection checklist is presented in Table 5. A check mark (✓) indicates that the corresponding inspection item applies to the target equipment. The inspection mainly involved checking instrument readings, operating status, and external condition. Based on these observations, the participants were required to determine whether the corresponding equipment was in a normal or abnormal condition. The inspection tasks primarily focused on the front-facing visible regions of the target equipment from the normal observation positions along the predefined route and did not involve circumferential or multi-angle inspection.

Each record was subsequently processed using the developed industrial patrol inspection visual attention analysis system, producing scores for each visual attention metric and an overall visual attention score. The quantitative scores are presented in Table 6. The S1–S7 metric identifiers used in Table 6 correspond to those defined in Table 1. The scores reported for the equipment-related visual attention metrics (S4–S7) are obtained by summing the corresponding scores across all target equipment items.

The overall visual attention scores of the inspection records ranged from 43.16 to 89.71, with an average of 75.99, as shown in Table 6. Records 2, 6, 11, and 16 achieved relatively high overall scores, reflecting relatively favorable visual attention patterns during these inspections. In contrast, Record 19 obtained an overall score of 43.16 and Record 20 obtained an overall score of 48.37, both substantially lower than the scores of the other records. Further analysis of the individual metric scores for Records 19 and 20 showed that the S2 scores were 9.75 for Record 19 and 9.73 for Record 20, indicating that there were substantial periods during the inspections in which no target equipment appeared in the video. Both records received 0 points for S4, indicating that the cumulative effective equipment observation durations generally failed to meet the prescribed thresholds. Both records received 3.75 points for S5, while the S6 scores were 2.50 for Record 19 and 1.25 for Record 20, reflecting low gaze stability and limited visual scanning coverage. In addition, Record 19 received 10.91 points for S3 and 1.25 points for S7, indicating that multiple target equipment items were unobserved and that few target equipment items were observed within the predefined maximum viewing distance.

**Figure 8:**
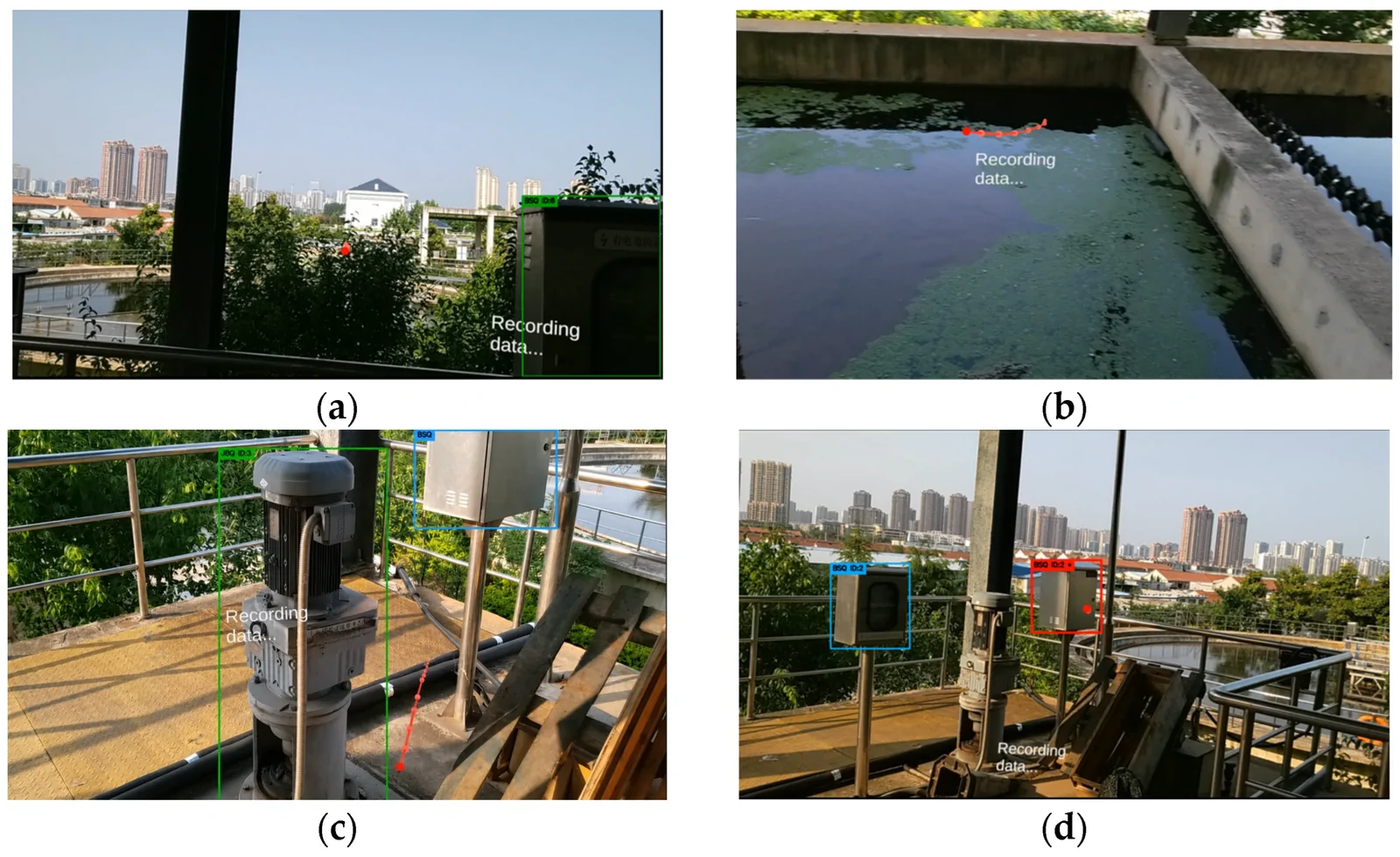
Representative visual attention patterns reflected by the visual attention metrics: (**a**) attention lapse in Record 3; (**b**) ineffective inspection interval in Record 6; (**c**) unobserved target equipment in Record 7; and (**d**) inappropriate viewing distance in Record 14.

To further verify whether the visual attention metric scores correspond to specific visual attention patterns during patrol inspection, four representative cases were selected from Records 3, 6, 7, and 14, as shown in Figure 8. Record 3 received an S1 score of 10, indicating that one attention-lapse event occurred during this inspection. As shown in Figure 8a, during the observation of one target equipment item in Record 3, the gaze point left the bounding box of the target equipment item and remained outside all target equipment bounding boxes for more than 4 s. Accordingly, the system identified one attention-lapse event. Record 6 received an S2 score of 11.18, indicating that its inspection process contained a certain proportion of ineffective inspection time. As shown in Figure 8b, during one interval in Record 6, the inspector kept the field of view directed toward the pool for an extended period, and no target equipment appeared in the frame. This interval was therefore classified as ineffective inspection time. Record 7 received an S3 score of 10.91, indicating that multiple target equipment items were unobserved during the inspection. As shown in Figure 8c, the system detected the mixer and identified an effective equipment observation segment for it; the mixer was therefore classified as observed and assigned an equipment ID. Although the transmitter on the right also appeared in the frame and was detected, the system did not identify an effective equipment observation segment for it; the transmitter was therefore classified as unobserved and was not assigned an equipment ID. Record 14 received an S7 score of 7.50, indicating that some equipment items were not observed from an appropriate distance. As shown in Figure 8d, although the gaze point was matched to the transmitter, the corresponding gaze-distance value exceeded the predefined maximum viewing distance of 1.5 m.

To provide an external reference for the quantitative visual attention results, the 21 inspection records were independently evaluated by three experts with extensive practical experience in industrial patrol inspection. Before the evaluation, the experts were instructed to review each inspection record as a whole and classify the visual attention reflected in the record as high (H), moderate (M), or low (L). The classification was based on an overall qualitative judgment of the visual attention reflected in each inspection record, mainly considering whether the prescribed target equipment received visual attention, whether the observations were sufficiently sustained, and whether obvious periods of attention disengagement or insufficient observation occurred during the inspection. The experts were not provided with the system-generated visual attention scores during the evaluation. They reviewed the synthesized inspection videos with overlaid equipment bounding boxes and 2D gaze points and independently assigned one of the three visual attention levels. The reference expert rating was determined based on the majority rating among the three experts, as shown in the “Majority” column in Table 7. For comparison with the expert ratings, the overall visual attention scores generated by the system were converted into the three predefined levels described in Section 3.5. The comparison between the system and expert ratings is presented in Table 7.

The system and reference expert ratings were consistent for 19 of the 21 inspection records, corresponding to an agreement rate of 90.5%. The two discrepancies occurred in Records 13 and 18, both of which were rated as M by the system and H by the reference expert rating. In addition, Spearman rank correlation was used to examine the association between the overall visual attention scores generated by the system and the ordinal reference expert ratings, with L, M, and H encoded as 1, 2, and 3. A strong positive correlation was observed $\left(\rho =0.839,\;p<0.001\right)$.

### 4.4. Discussion

The eye-tracking experiment demonstrated the feasibility of acquiring eye-tracking data during on-site patrol inspection and projecting the resulting 3D gaze points onto the corresponding first-person video frames to support gaze–equipment matching. The YOLO12m model achieved satisfactory detection performance on the independent test set, providing a basis for locating target equipment in the inspection videos. The visual attention scores of the 21 inspection records ranged from 43.16 to 89.71, showing clear differences among the records. The analysis of Records 19 and 20 indicated that their low scores resulted from the combined effects of multiple visual attention patterns rather than a single metric, while the representative cases showed that the individual metrics could reflect specific patterns such as attention-lapse events, ineffective inspection intervals, unobserved target equipment, and inappropriate viewing distances. In addition, the system and reference expert ratings were consistent for 19 of the 21 records, and the system-generated visual attention scores were strongly correlated with the reference expert ratings $\left(\rho =0.839,\;p<0.001\right)$. These results suggest that, under the present experimental conditions, the proposed method can quantitatively analyze the visual attention of inspectors toward target equipment and provide interpretable results.

Several limitations should be considered when interpreting these results. First, the visual attention metrics, parameter settings, and score allocation were determined based on expert consultation, preliminary inspection trials, and the requirements of the present inspection task, but their sensitivity to alternative parameter values has not been systematically analyzed. Therefore, the proposed criteria should be regarded as a task-specific quantitative scheme under the present experimental conditions rather than a universal standard for industrial inspection. Second, the reported object detection performance was evaluated using individual images from the independent test set and does not directly represent temporal detection stability during continuous video processing. Although cross-frame association and short-term retention of the most recent valid detection boxes for up to 0.2 s were used to reduce the influence of brief detection dropouts, the effects of detection errors and temporal instability on the S1–S7 scores have not been quantified through comparison with manually annotated inspection videos. Third, the accuracy of nearest-neighbor temporal alignment and 3D-to-2D gaze point projection was not quantitatively evaluated. Residual temporal mismatch, rapid head movements, and eye-tracking errors may cause deviations in the projected gaze points, particularly when a gaze point is close to the boundary of an equipment detection box. Fourth, the experiment included 10 participants and 21 inspection records collected at one industrial site. Although the selected targets include several equipment types commonly used in industrial facilities, the current evidence remains limited with respect to other participants, sites, and operating conditions. Finally, the comparison with the expert ratings should be regarded as preliminary validation because the experts evaluated synthesized videos containing equipment bounding boxes and projected 2D gaze points generated by the proposed system. Their judgments may therefore have been influenced by target-equipment detection or gaze-point projection errors. Accordingly, the expert evaluation supports the three-level classification under the present experimental conditions but does not fully validate the 100-point scale or each individual S1–S7 metric.

## 5. Conclusions

This study proposes an industrial patrol inspection visual attention analysis method integrating eye tracking and deep learning-based object detection. The method associates eye-tracking data with object detection results, thereby enabling quantitative characterization of the visual attention of inspectors toward target equipment. The experimental results demonstrate that, under the present experimental conditions, the proposed method can quantitatively analyze the visual attention of inspectors toward target equipment during patrol inspection using multiple metrics and provide interpretable results.

Future work will further refine the visual attention metrics, parameter settings, and score allocation through sensitivity analyses. It will also evaluate video-level detection accuracy and temporal stability using manually annotated detections, quantify temporal alignment and 3D-to-2D gaze point projection accuracy, and conduct experiments with more participants across different industrial sites and operating conditions to further assess the applicability of the method and the practical effects of using the system on inspectors. Further validation of the 100-point scoring scheme and the individual S1–S7 metrics will also be conducted.

## Figures and Tables

**Table 1:** Visual attention metrics and score allocation.

No.	Visual Attention Metric	Score Allocation
S1	Inspection Attentiveness	15 points
S2	Effective Inspection Time Ratio	15 points
S3	Target Equipment Observation Rate	15 points
S4	Equipment Observation Duration Compliance	1/4 of the score allocated to each equipment item
S5	Gaze Stability	1/4 of the score allocated to each equipment item
S6	Visual Scanning Coverage	1/4 of the score allocated to each equipment item
S7	Viewing Distance Appropriateness	1/4 of the score allocated to each equipment item

**Table 2:** Number of annotated bounding boxes for each target equipment class.

Class ID	Class Abbreviation	Equipment Class	Number of Annotated Bounding Boxes
0	FM	Valve	98
1	BSQ	Transmitter	684
2	JBQ	Mixer	289
3	LLJ	Flowmeter	162
4	GNJ	Sludge Scraper	162

**Table 3:** Class-specific detection performance of the YOLO12m model on the independent test set.

Equipment Class	Instances	Precision	Recall	AP@0.5
Valve (FM)	16	0.9605	1.0000	0.9950
Transmitter (BSQ)	128	0.9303	0.9387	0.9773
Mixer (JBQ)	52	0.9555	0.9808	0.9909
Flowmeter (LLJ)	27	0.8924	0.9630	0.9825
Sludge Scraper (GNJ)	38	0.9668	0.9737	0.9493

**Table 4:** Participant experience levels and corresponding inspection records.

Participant	Experience Level	Records
P1	High	2, 11
P2	Moderate	6, 13
P3	Moderate	15, 17
P4	Moderate	16, 18
P5	Low	1, 5
P6	Low	3, 7
P7	Low	4, 9
P8	Low	8, 14
P9	Low	10, 19
P10	Low	12, 20, 21

**Table 5:** Inspection checklist for the target equipment.

No.	Target Equipment	Instrument Reading	Operating Status	External Condition
1	Valve		✓	✓
2	Transmitter	✓	✓	✓
3	Mixer		✓	✓
4	Transmitter	✓	✓	✓
5	Flowmeter	✓	✓	✓
6	Transmitter	✓	✓	✓
7	Mixer		✓	✓
8	Transmitter	✓	✓	✓
9	Transmitter	✓	✓	✓
10	Sludge Scraper		✓	✓
11	Transmitter	✓	✓	✓

**Table 6:** Quantitative visual attention scores for the inspection records.

Record	S1	S2	S3	S4	S5	S6	S7	Overall Score
1	15.00	12.86	15.00	8.75	5.00	8.75	8.75	74.11
2	15.00	13.57	13.64	12.50	11.25	11.25	12.50	89.71
3	10.00	12.35	15.00	12.50	10.00	10.00	10.00	79.85
4	15.00	13.02	13.64	11.25	8.75	8.75	8.75	79.16
5	10.00	12.88	12.27	8.75	11.25	5.00	10.00	70.15
6	15.00	11.18	15.00	13.75	8.75	12.50	11.25	87.43
7	15.00	13.23	10.91	8.75	6.25	7.50	7.50	69.14
8	15.00	12.52	12.27	11.25	6.25	7.50	8.75	73.54
9	15.00	12.11	12.27	10.00	7.50	10.00	6.25	73.13
10	10.00	12.88	15.00	8.75	8.75	11.25	8.75	75.38
11	15.00	11.12	15.00	12.50	8.75	13.75	11.25	87.37
12	15.00	12.71	15.00	10.00	7.50	10.00	7.50	77.71
13	15.00	12.34	15.00	11.25	7.50	12.50	10.00	83.59
14	10.00	13.06	12.27	10.00	7.50	8.75	7.50	69.08
15	15.00	12.63	15.00	12.50	8.75	10.00	8.75	82.63
16	15.00	12.61	15.00	13.75	12.50	8.75	10.00	87.61
17	15.00	12.53	13.64	12.50	12.50	6.25	10.00	82.42
18	15.00	12.53	13.64	11.25	11.25	8.75	10.00	82.42
19	15.00	9.75	10.91	0.00	3.75	2.50	1.25	43.16
20	15.00	9.73	13.64	0.00	3.75	1.25	5.00	48.37
21	15.00	12.33	15.00	6.25	13.75	10.00	7.50	79.83

**Table 7:** Comparison between system and expert ratings for the inspection records.

Record	Overall Score	System	Expert 1	Expert 2	Expert 3	Majority
1	74.11	M	M	M	M	M
2	89.71	H	H	H	H	H
3	79.85	M	M	M	M	M
4	79.16	M	M	M	M	M
5	70.15	M	M	M	M	M
6	87.43	H	H	H	H	H
7	69.14	M	M	M	M	M
8	73.54	M	M	M	M	M
9	73.13	M	M	M	M	M
10	75.38	M	M	M	M	M
11	87.37	H	H	H	H	H
12	77.71	M	M	M	M	M
13	83.59	M	H	H	M	H
14	69.08	M	M	M	M	M
15	82.63	M	H	M	M	M
16	87.61	H	H	H	H	H
17	82.42	M	M	M	H	M
18	82.42	M	M	H	H	H
19	43.16	L	L	L	L	L
20	48.37	L	L	L	L	L
21	79.83	M	M	M	M	M

## Data Availability

The data presented in this study are openly available in GitHub Releases at https://github.com/dwq0117-sys/industrial-inspection-visual-attention-dataset/releases/tag/v1.0.0 (accessed on 8 September 2026).
